# Predictive value of CPS combined with inflammatory markers for pathological remission of locally advanced head and neck squamous cell carcinoma after adjuvant immunochemotherapy

**DOI:** 10.3389/fmolb.2025.1593742

**Published:** 2025-05-01

**Authors:** Yudong Ning, Han Li, Yixuan Song, Yuqin He, Shaoyan Liu, Yang Liu

**Affiliations:** Department of Head and Neck Surgery, National Cancer Center/National Clinical Research Center for Cancer/Cancer Hospital, Chinese Academy of Medical Sciences and Peking Union Medical College, Beijing, China

**Keywords:** pathological complete response, neoadjuvant immunotherapy, head neck squamous carcinoma, combined positive score, pathological remission, immune checkpoint inhibitors, pathological tumor response, neutrophil to platelet count ratio

## Abstract

**Objective:**

To explore the predictive value of the combined positive score (CPS) and the neutrophil-to- platelet count ratio (NPR) for surgical pathological remission in patients with locally advanced head and neck squamous cell carcinoma (LAHNSCC) who have undergone neoadjuvant immunotherapy combined with chemotherapy (NICC).

**Method:**

Patients with LAHNSCC who underwent NICC and surgery from May 2021 to September 2023 were retrospectively analyzed. CPS, NPR and other clinically relevant parameters were collected, which includes gender, age, tumor types, multiple cancer, differentiation, T staging, N staging, immunotherapy cycles and postoperative pathological remission degree.

**Result:**

Patients with a higher CPS were significantly associated with a higher pathological complete response (PCR) of the primary site (PPCR) (P = 0.034) and a higher PCR of the lymph nodes (LPCR) (P = 0.085). Specifically, patients with a CPS of ≥20 demonstrated a higher rate of severe pathologic tumor response (PTR), with values of 80.8% compared to 66.7% and 50%. Notably, even patients with a CPS <1 had a relatively high severe PTR rate of 66.7%. Moreover, patients with NPR <0.024 exhibited a higher severe PTR, regardless of the CPS subgroups (P < 0.05).

**Conclusion:**

Higher CPS can be considered a good predictor of higher PCR after NICC in patients with LAHNSCC. Patients with CPS <1 can still achieve a higher PTR. Patients with NPR <0.024 can help achieve a higher severe PTR in patients with LAHNSCC regardless of the CPS.CPS combined with NPR may have a better predicted value for surgical PTR of HNSCC after NICC.

## Introduction

Head and neck squamous cell carcinoma (HNSCC) is a common type of malignant tumor. Currently, surgery and radiotherapy serve as the mainstay treatments for patients with HNSCC. Regrettably, a significant number of patients are diagnosed at the intermediate or advanced stages. Even after undergoing a combination of comprehensive treatments such as surgery, radiotherapy, and chemotherapy, the recurrence rate and mortality rate remain remarkably high. Immunotherapy has emerged as a rapidly evolving cancer treatment modality in recent years. Tumors are capable of evading the immune system by expressing immune checkpoint ligands, including programmed death receptor-1 (PD-1), programmed death ligand-1 (PD-L1), and cytotoxic T-lymphocyte-associated protein 4 (CTLA-4). Based on several clinical trials, such as EYNOTE-012, KEYNOTE-040, KEYNOTE-048, and CheckMate141, it has been reported that immune checkpoint inhibitors (ICIs), specifically Nivolumab and Pembrolizumab, were approved by the U.S. Food and Drug Administration (FDA) in 2016. These drugs have become novel treatment options for recurrent/metastatic HNSCC, primarily because of their efficacy in prolonging overall survival (Seiwert et al., 2016; Cohen et al., 2019; Harrington et al., 2017; Burtness et al., 2019).Based on these positive results, many centers have investigated neoadjuvant immunotherapy for locally advanced HNSCC and achieved satisfactory results (Leidner et al., 2021; Ferris et al., 2021; Vos et al., 2021; Ning et al., 2025).

Recent clinical trials have indicated that not all patients are ideal candidates for immunotherapy. Consequently, identifying the population that can benefit most from a favorable immune response has emerged as a crucial research priority. In previous investigations, the combined positive score (CPS) has been employed as a valuable tool for assessing the efficacy of immunotherapy. Nevertheless, the majority of these earlier studies primarily focused on patients diagnosed with recurrent or metastatic HNSCC. There have been relatively few studies exploring the predictive value of CPS in locally advanced head and neck squamous cell carcinoma (LAHNSCC) patients undergoing neoadjuvant therapy.

Moreover, the determination of CPS relies on the detection of PD-L1 within the tumor microenvironment, necessitating the acquisition of tumor tissue samples. Tumors are known to exhibit substantial intra-tumoral heterogeneity, and PD-L1 is a dynamic biomarker. These factors imply that CPS might have certain inherent limitations. As such, there is an urgent demand for biomarkers that are easy to utilize, reliable, and cost-effective to accurately predict how HNSCC patients will respond to neoadjuvant chemoimmunotherapy. The tumor microenvironment is regulated by the inflammatory immune system (Turan et al., 2018), and platelet count is also closely related to tumor development (Peila and Rohan, 2020; Ning et al., 2021).Based on the data from our center, we conducted a study to investigate the predictive value of CPS and neutrophil to platelet count ratio (NPR) for the surgical pathological remission (PR) of patients with HNSCC after undergoing neoadjuvant immunotherapy combined with chemotherapy (NICC),in order to provide better guidance in clinical practice.

### Characteristics of the cohort

A retrospective analysis was performed on a cohort of 41 patients with LAHNSCC who were treated by NICC followed by surgery in the Head and Neck Surgery department of National Cancer Hospital from May 2021 to September 2023. The data on gender, age, tumor types, differentiation, multiple cancer, T staging, N staging, immunotherapy cycles, CPS, NPR and PR were collected. The CPS data was divided into 3 groups based on the cutoff of 1% and 20%, including CPS <1, 1 ≤ CPS <20, and CPS ≥20, according to KEYNOTE-048 (Burtness et al., 2022; Yilmaz et al., 2023a). PR included pathological tumor response (PTR) (mild, moderate, and severe) and pathological complete response (PCR). PCR included PCR of the primary site (PPCR), PCR of the lymph node (LPCR), and PCR of the primary site and lymph node (PLPCR). T staging and N staging were performed based on the eighth edition of staging. The clinical information of all patients is well documented.

### Diagnostic evaluation

All patients underwent a biopsy of the primary sites before NICC and their cancer was pathologically confirmed as squamous cell carcinoma with or without the biopsy of the lymph node. All patients were evaluated using laryngoscopy, enhanced computerized tomography (CT), and enhanced magnetic resonance imaging (MRI) before treatment to determine the clinical stage.

All postoperative pathologies were evaluated by experienced pathologists. The CPS value is defined as the sum of PD-L1-stained tumor cells and tumor-associated immune numbers per 100 tumor cells. CPS were reported based on the biopsy of the primary sites. PCR is defined as the absence of tumor cells in surgically removed tissue. PTR is quantified based on the proportion of tumor necrosis, keratin fragments, and giant cells/histiocytes in the surgically removed tissue as mild PTR (0%–20%), moderate PTR (20%–80%), and severe PTR (80%–100%).

### Treatment method

All patients were treated by NICC followed by surgery. NICC included PD-1 immune checkpoint inhibitors (ICIs) (Pabolizumab, Toripalimab, Tislelizumab, or Sindillimab) with cisplatin and taxol × 1, 2, 3, or 4 cycles.

### Statistic method

Chi-square test was used to compare the correlation of three subgroups of CPS in predicting PR. when a contingency table has an expected count of 5 or fewer in 20% or more of the cells, Fisher’s exact test should be used.

The Receiver Operating Characteristic (ROC) curve evaluated the predictive value of NPR for severe PR, and looked for the cutoff value.The predictive value of NPR based on cutoff calve for severe PR was analyzed using univariate and multivariate analysis.Chi-square test compared the value and correlation analysis of NPR and CPS subgroup in predicting severe PTR. when a contingency table has an expected count of 5 or fewer in 20% or more of the cells, Fisher’s exact test should be used. And a multi-factor analysis of variance was adopted. P < 0.05 indicates there was statistical significance. All statistics were performed by SPSS.27.

## Result

### Patient characteristics

**FIGURE 1:**
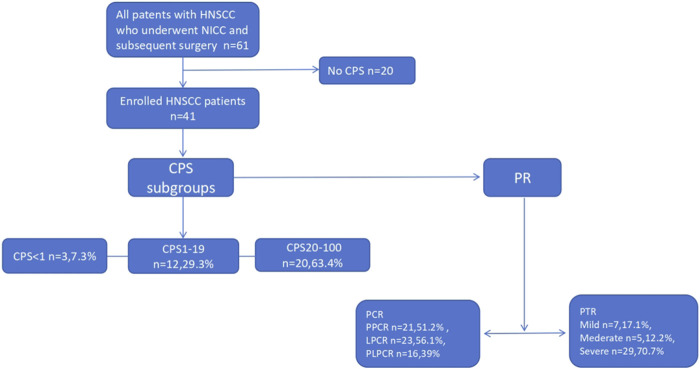
Flow chat. PR: pathological remission PTR: pathological tumor response, PCR: pathological complete response, PLPCR: pathological complete response of the primary site and the lymph node, PPCR: pathological complete response of the primary site,PLPCR: pathological complete response of the lymph node, ICIs: immune checkpoint inhibitors, CPS: combined positive score, LAHNSCC: Locally advanced head and neck squamous cell carcinoma,HNSCC: head and neck squamous cell carcinoma; NICC: neoadjuvant immunotherapy combined with chemotherapy (NICC).

As shown in Table 1 and Figure 1, 41 patients with HNSCC were included this study. The study patients were assigned to two groups based on their gender or age (age: 60 years) and into four groups according to the tumor types, including oropharyngeal cancer (13, 32.7%), hypopharyngeal cancer (24, 58.5%), oral cancer (3,7.3%) and laryngeal cancer (1,2.4%). The number of multiple cancer in this cohort is 5 (12.2%). The differentiation for this cohorts were high (6, 14.6%), middle (23, 56.1%), and low (12, 29.3%). According to the eighth edition TNM staging system, the patients distributed in Tis, T1, T2, T3, and T4a were 1 (2.4%), 5 (12.2%), 16 (39%), 6 (14.6%), and 13 (31.7), respectively. The corresponding numbers of patients in N1, N2a, N2b, N2c, N3a, and N3b were 3 (7.3%), 2 (4.9%), 24 (58.5), 7 (17.1%), 0 and 5 (12.2%), respectively. The patients were assigned to 4 groups by immunotherapy cycles, including 1 (2, 4.9%), 2 (31, 75.6%), 3 (7, 17.1%), and 4 (1,2.4%), and 4 groups by ICIs including pabolizumab 27 (65.9%), Toripalimab 4 (9.8%), Tislelizumab 8 (10.5%), and Sintilimab 2 (4.9%). CPS was divided into 3 groups by the cutoff of 1% and 20%, including CPS <1 (3.7.3%), 1 ≤ CPS <20 (12.29.3), CPS ≥20 (26, 63.4). Mild, moderate, and severe PTR were 7 (17.1%), 5 (12.2%), and 29 (70.7%). PCR were evaluated by 3 groups, which included PPCR (21, 51.2%), LPCR (23, 56.1%), and PLPCR (16, 39.0%).

**TABLE 1:** Characteristics of the cohort and the association of PCR with clinical parameters.

Clinical characters	Number (%)
Gender Female	1 (2.4)
Male	40 (97.6)
Age (years old) ≥60	21 (51.2)
<60	20 (48.8)
Tumor Types Oropharyngeal cancer	13 (31.7)
Hypopharyngeal cancer	24 (58.5)
Oral cancer	3 (7.3)
Laryngeal cancer	1 (2.4)
Multiple Cancer No	36 (87.8)
Yes	5 (12.2)
Differentiation	
High	6 (14.6)
Mid	23 (56.1)
Poor	12 (29.3)
T Staging Tis	1 (2.4)
T1	5 (12.2)
T2	16 (39)
T3	6 (14.6)
T4a	13 (31.7)
N Saging	
N1	3 (7.3)
N2a	2 (4.9)
N2b	24 (58.5)
N2c	7 (17.1)
N3a	0 (0)
N3b	5 (12.2)
Immunotherapy cycle	
1	2 (4.9)
2	31 (75.6)
3	7 (17.1)
4	1 (2.4)
ICIs types	
Pabolizumab	27 (65.9)
Toripalimab	4 (9.8)
Tislelizumab	8 (10.5)
Sintilimab	2 (4.9)
CPS	
<1	3 (7.3)
2–19	12 (29.3)
20–100	26 (63.4)
PTR	
Mild	7 (17.1)
Moderate	5 (12.2)
Severe	29 (70.7)
PPCR	
yes	21 (51.2)
No	20 (48.8)
LPCR	
yes	23 (56.1)
No	18 (43.9)
PLPCR	
yes	16 (39.0)
No	25 (61)
Total	41 (100)

PCR, pathological complete response; PLPCR, pathological complete response of the primary site and the lymph node, PPCR, pathological complete response of the primary site; PLPCR, pathological complete response of the lymph node; PTR, pathological tumor response; ICIs, immune checkpoint inhibitors; CPS, combined positive score.

### The predictive value of CPS for PR of LAHNSCC after NICC

In Table 2, to exclude the influence of other clinical parameters, we compared the correlation between the CPS subgroups and other clinical parameters. Based on our results, the baseline of other clinical parameters in the CPS subgroups was consistent, with no statistically significant difference.

**TABLE 2:** The baseline of CPS subgroups.

Clinical characters	CPS (<1) (%)	CPS (2–19)(%)	CPS (20–100)(%)	P Value
Gender Female	0 (0)	0 (0)	1 (100)	0.744
Male	3 (7.5)	12 (30.0)	25 (62.5)	
Age (years old) ≥60	2 (9.5)	8 (38.1)	11 (52.4)	0.323
<60	1 (5.0)	4 (20.0)	15 (75.0)	
Tumor Type Oropharyngeal cancer	1 (7.7)	2 (15.4)	10 (76.9)	0.287
Hypopharyngeal cancer	1 (4.2)	10 (41.7)	13 (54.2)	
Oral cancer	1 (33.3)	0 (0)	2 (66.7)	
Laryngeal cancer	0 (0)	0 (0)	1 (100)	
Multiple Cancer No	3 (8.3)	9 (25.0)	24 (66.7)	0.253
Yes	0 (0)	3 (60.0)	2 (40.0)	
Differentiation				
High	0 (0)	1 (16.7)	5 (83.3)	0.182
Mid	2 (8.7)	10 (43.5)	11 (47.8)	
Poor	1 (8.3)	1 (8.3)	10 (83.3)	
T Staging Tis	1 (100)	0.198	1 (100)	0.457
T1	3 (60.0)		3 (60.0)	
T2	11 (68.8)		11 (68.8)	
T3	2 (33.3)		2 (33.3)	
T4a	4 (30.8)		6 (46.2)	
N Saging				
N1	2 (66.7)	0.659	2 (66.7)	0.415
N2a	1 (50.0)		2 (100)	
N2b	11 (45.)		14 (58.3)	
N2c	3 (42.9)		2 (28.6)	
N3a	0 (0)		0 (0)	
N3b	4 (80.0)		3 (60.0)	
Immunotherapy cycle				
1	1 (50.0)	0.147	1 (50.0)	0.841
2	13 (41.9)		17 (54.8)	
3	6 (85.7)		4 (57.1)	
4	1 (100)		1 (100)	
ICIs types				
Pabolizumab	1 (3.7)	8 (29.6)	18 (66.7)	0.298
Toripalimab	0 (0)	1 (25.0)	3 (75.0)	
Tislelizumab	1 (12.5)	3 (37.5)	4 (50.0)	
Sintilimab	1 (50.0)	0 (0)	1 (50.0)	

**FIGURE 2:**
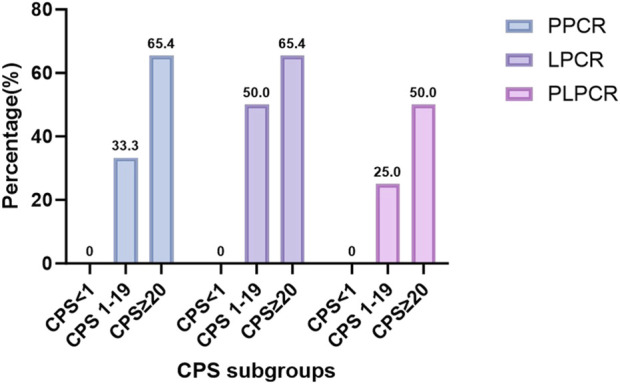
The correlation between CPS subgroups and PCR: Higher CPS has a higher PCR(P < 0.05) PCR: pathological complete response, PLPCR: pathological complete response of the primary site and the lymph node, PPCR: pathological complete response of the primary site,LPCR: pathological complete response of the lymph node, CPS: combined positive score.

**FIGURE 3:**
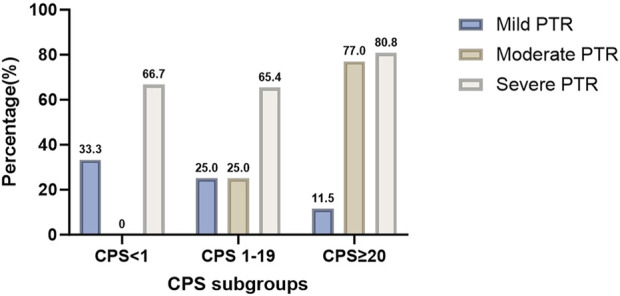
The correlation between CPS subgroups and PTR (P > 0.05).Patients with CPS ≥20 had a higher severe PTR. Even patients with CPS <1 still had a higher severe PTR (66.7%). PR: pathological remission, PTR: pathological tumor response, CPS: combined positive score.

ICIs, immune checkpoint inhibitors; CPS, combined positive score.

(P > 0.05). As shown in Table 3 and Figure 2, the patients with higher in the CPS groups and significantly associated with higher PPCR (65.4% > 33.3% > 0, P = 0.034), LPCR (65.4% > 33.3 > 0, P = 0.085 < 0.1). About PLPCR, patients with higher CPS showed a higher PLPCR (50% > 25% > 0, P = 0.121). CPS showed no significant association with PTR (P = 0.299), but patients with CPS ≥20 showed a higher severe PTR (80.8 vs. 66.7,50). Even patients with CPS <1 showed a higher severe PTR (66.7%) (Figure 3).

**TABLE 3:** The association of CPS subgroups with PR and PCR.

Clinical characters	CPS (<1)	CPS (2–19)	CPS (20–100)	P Value
PTR (%) Mild	1 (33.3)	3 (25.0)	3 (11.5)	0.299
Moderate	0 (0)	3 (25.0)	2 (7.7)	
Severe	2 (66.7)	6 (65.4)	21 (80.8)	
PPCR(%)				
YES	0 (0)	4 (33.3)	17 (65.4)	0.034
NO	3 (100)	8 (66.7)	9 (34.6)	
LPCR(%)				
YES	0 (0)	6 (50.0)	17 (65.4)	0.085
NO	3 (100)	6 (50.0)	9 (34.6)	
PLPCR(%)				
YES	0 (0)	3 (25.0)	13 (50.0)	0.121
NO	3 (100)	9 (75.0)	13 (50.0)	

PCR, pathological complete response; PLPCR, pathological complete response of the primary site and the lymph node, PPCR, pathological complete response of the primary site; PLPCR, pathological complete response of the lymph node; PTR, pathological tumor response; ICIs, immune checkpoint inhibitors; CPS, combined positive score.

### The association of clinical characteristics with severe PTR

**FIGURE 4:**
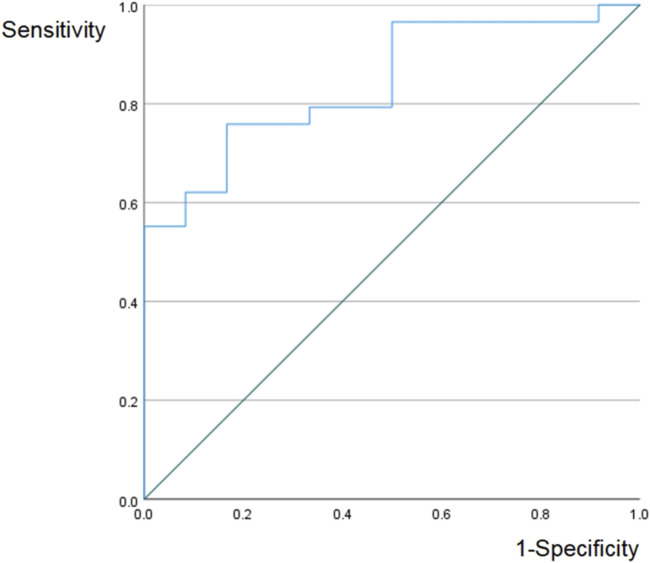
ROC curve NPR had good predicted value for severe PTR (AUC = 0.842 P = 0.01 cutoff = 0.024). PTR: pathological tumor response, NPR: neutrophil to platelet count ratio, ROC receiver operating characteristic, AUC: area under curve.

As shown in Figure 4, NPR with the cutoff of 0.024 had good predicted value for severe PTR (P = 0.01). In Table 4, we analyzed the association of clinical characteristics with severe PTR. By univariate and multivariate analysis, patients with age <60 years old had significantly higher severe PTR (90% VS 52.4%, P < 0.05), and patients with NPR <0.024 had significantly higher severe PTR (75.9% VS 41.2%, P < 0.05). Furthermore, we combine NPR and CPS to predict severe PTR in Table 5. In group of CPS 20–100, patients with NPR <0.024 could achieve a higher severe PTR (100% VS 54.5%, P = 0.004). In group of CPS 2–19, patients with NPR <0.024 still had a significantly higher severe PTR (71.4% VS 20.0%). Even CPS <1, patients with NPR <0.024 still had a higher severe PTR.

**TABLE 4:** The association of clinical characteristics with severe PTR.

Clinical characters	Number (%)	Severe PR (100%)	Univariate analysis P Value	Multivariate analysis P Value
Gender Female	1 (2.4)	1 (100)	0.515	0.993
Male	40 (97.6)	28 (70.0)		
Age (years old) ≥60	21 (51.2)	11 (52.4)	0.008	0.007
<60	20 (48.8)	18 (90.0)		
Tumor Types Oropharyngeal cancer	13 (31.7)	11 (84.6)	0.199	0.975
Hypopharyngeal cancer	24 (58.5)	14 (58.3)		
Oral cancer	3 (7.3)	3 (100)		
Laryngeal cancer	1 (2.4)	1 (100)		
Multiple Cancer No	36 (87.8)	26 (72.2)	0.574	0.763
Yes	5 (12.2)	3 (60.0)		
Differentiation				
High	6 (14.6)	6 (100)	0.184	0.710
Mid	23 (56.1)	16 (69.6)		
Poor	12 (29.3)	7 (58.3)		
T State Tis	1 (2.4)	1 (100)	0.516	0.206
T1	5 (12.2)	4 (80)		
T2	16 (39)	13 (81.3)		
T3	6 (14.6)	3 (50)		
T4a	13 (31.7)	8 (61.5)		
N Sate				
N1	3 (7.3)	2 (66.7)	0.536	0.924
N2a	2 (4.9)	1 (50)		
N2b	24 (58.5)	17 (70.8)		
N2c	7 (17.1)	4 (57.1)		
N3a	0 (0)	0		
N3b	5 (12.2)	5 (100)		
Immunotherapy cycle				
1	2 (4.9)	2 (100)	0.14	0.271
2	31 (75.6)	19 (61.3)		
3	7 (17.1)	7 (100)		
4	1 (2.4)	1 (100)		
ICIs types				
Pabolizumab	27 (65.9)	19 (70.4)	0.630	0.671
Toripalimab	4 (9.8)	2 (50)		
Tislelizumab	8 (10.5)	6 (75)		
Sintilimab	2 (4.9)	2 (100)		
NPR				
≥0.024	17 (41.5)	7 (41.2)	<0.01	<0.01
<0.024	24 (58.5)	22 (75.9)		
Total	41 (100)	29 (70.7)		

ICIs, immune checkpoint inhibitors; PTR, pathological tumor response; NPR, neutrophil to platelet count ratio.

**TABLE 5:** The association of NPR with severe PTR based on CPS subgroups.

Clinical characters	Number (%)	Severe PR (100%)	P Value
CPS(20–100) ≥0.024	11 (42.3)	6 (54.5)	0.004
<0.024	15 (57.7)	15 (100)	
CPS(2–19) ≥0.024	5 (51.2)	1 (20.0)	-
<0.024	7 (48.8)	5 (71.4)	-
CPS(<1) ≥0.024	1 (33.3)	0	-
<0.024	2 (66.7)	2 (100%)	-
Total	41 (100)	29 (70.7)	

ICIs, immune checkpoint inhibitors; PTR, pathological tumor response; NPR, neutrophil to platelet count ratio.

## Discussion

Immunohistochemical staining indicated that PD-L1 was expressed on both tumor cells and immune cells. The clinical benefits of immune checkpoint inhibitors were associated with the expression of PD-L1 on immune cells other than tumor cells, thus generating the CPS. CPS is defined as the addition of the number of PD-L1-stained cells (including tumor cells, lymphocytes, and macrophages) divided by the total number of living tumor cells, and multiplying by 100. CPS is expressed as a number and not a percentage. Although the result can exceed 100, the maximum score is defined as a CPS of 100. CPS was initially used in the immunotherapy of LAHNSCC, including KEYNOTE-012 (Stage I) (Seiwert et al., 2016) and KEYNOTE-055 (Stage II) (Bauml et al., 2017). KEYNOTE-012 is an open-label, multi-cohort, multi-center phase I study that evaluated the safety and clinical efficacy of pbolizumab (an ICI) in the treatment of HNSCC. KEYNOTE-012 results indicated that CPS is significantly associated with clinical benefits. Patients showing positive PD-L1 expression on the CPS had a higher PR than those showing negative PD-L1 expression (21% vs. 6%, P = 0.023), and a greater statistical difference in PFS and overall survival (OS) was found between the two groups. Keymat-055 is a Phase II, single-arm study that evaluated the efficacy and safety of pembrolizumab therapy in recurrent/metastatic HNSCC resistant to platinum and cetuximab. KEYNOTE-055 results indicated that the higher the CPS, the higher the objective response rate. The objective response rate for CPS ≥50 was as high as 27%, whereas the objective response rate for CPS ≥1 was only 18%. Several studies have shown the clinical use of CPS stratification in digestive tract tumors and other cancer types (Kojima et al., 2020; Shitara et al., 2020; Chao et al., 2021; Janjigian et al., 2021).

The KEYMAT-048 study that was published in the Lancet in 2019 (Burtness et al., 2019) summarized the data on OS, progression-free survival (PFS), and adverse events from a phase 3 randomized clinical study. This study included patients with recurrent/metastatic HNSCC with a CPS of ≥20 and a CPS of ≥1. The clinical outcomes were compared with palizizumab alone or combined chemotherapy vs cetuximab combined chemotherapy. This result showed pabolizumab combined with chemotherapy can obtain better OS in patients with CPS ≥20 and CPS ≥1,which changed the present first-line treatment landscape of recurrent/metastatic HNSCC. Therefore, whether palibrizumab or combination chemotherapy can be an option for patients with recurrent/metastatic HNSCC with CPS <1 and CPS 1–19 should be investigated. Data from the 2022 Keynote-048 study for patients with recurrent metastatic HNSCC with CPS <1 and CPS 1–19 were made public (Burtness et al., 2022). KEYNOTE-048 concluded that with the increase of PD-L1 expression in patients, the efficacy of palizizumab alone and palizizumab combined with chemotherapy increases. For patients with CPS 1–19, the results generally support previous findings. In the CPS 1–19 population, palizizumab combined with chemotherapy showed a significant median OS benefit compared with cetuximab combined with chemotherapy. For patients with low PD-L1 expression, CPS <1, it is still necessary to find a better treatment. Asco guideline of 2022 (Yilmaz et al., 2023a; Yilmaz et al., 2023b) reported the following recommendation:

1. PD-L1 with CPS ≥1 should be interpreted as positive and correlate with a clinical benefit to PD-1 inhibitors.
2. Pembrolizumab monotherapy or pembrolizumab, platinum, and fluorouracil should be used as first-line treatment for patients with recurrent or metastatic HNSCC with a CPS of ≥1.
3. Pembrolizumab, platinum, and fluorouracil should be offered as first-line treatment for patients with recurrent or metastatic HNSCC with a CPS <1.
4. Pembrolizumab or nivolumab should be offered to patients with platinum-refractory recurrent or metastatic HNSCC, regardless of CPS status.

However, few studies have been performed on the predicted value of CPS for advanced HNSCC through NICC.

In our study, all patients were treated by NICC followed by surgery. NICC included PD-1 ICIs (Pabolizumab, Toripalimab, Tislelizumab, and Sindillimab), cisplatin, and taxol × 1, 2, 3, or 4 rounds. We found that patients with higher CPS groups were significantly associated with higher PPCR (65.4% > 33.3% > 0, P = 0.034), and LPCR (65.4% > 33.3 > 0, P = 0.085 < 0.1). Regarding PLPCR, patients with higher CPS had a higher PLPCR (50% > 25% > 0, P = 0.121) (Table 3; Figure 2). CPS had no significant association with PTR (P = 0.299), but patients with CPS ≥20 had a higher severe PTR (80.8 vs. 66.7, 50). Even patients with CPS <1 had a higher severe PTR (66.7%) (Figure 3). Higher CPS can be a good predictor of higher PCR after NICC for LAHNSCC. Patients with CPS <1 can achieve a higher PR. Due to the dynamic interactions between these antibodies and the immune microenvironment and the variability of the immune environment in different tumor types, the predictability of PD-L1 expression remains uncertain and contradictory (Burtness et al., 2019; Doroshow et al., 2021; Topalian et al., 2012; Shen and Zhao, 2018; Yu et al., 2019). Perhaps because of the spatial heterogeneity of the tumor, the expression of PD-L1 within the tumor and between the primary and metastatic sites may be quite heterogeneous, so the tissue biopsy at a single site cannot well represent the overall tumor status of the patient. In addition, some circulating tumor cells and exosomes in peripheral blood also have partial PD-1 expression. So CPS may be inaccurate.

Also, the immune status of the body receiving the initial treatment is inconsistent with that after the relapse and metastasis treatment. Our results also suggest that CPS may not be so accurate and that more predictors of immunotherapy efficacy should be sought, especially for severe PTR. When it comes to the role of PD-L1 testing in HNSCC, several crucial issues need to be taken into account (Taverna and Franchi, 2023). The first significant aspect pertains to the reproducibility of the staining protocol utilized for the immunohistochemical assessment of PD-L1 expression. The expression levels and staining distributions can vary substantially depending on the different protocols in use. This variability limits the ability to compare data obtained from different research centers. On the other hand, different observers may arrive at different interpretations of the results. Several studies have been conducted to evaluate the consistency of different staining protocols in assessing PD-L1 expression in HNSCC. These studies have also examined the inter-observer variability in evaluating the outcomes (Hodgson et al., 2018; de Ruiter et al., 2021; Crosta et al., 2021; Cerbelli et al., 2022). Overall, there was moderate to significant agreement between the different PD-L1 assays, as well as inconsistencies between observers, especially when the assessment was performed by a trained pathologist.

In 1863, Rudolf Virchow first posited that chronic inflammation significantly impacts the onset and progression of tumors (Balkwill and Mantovani, 2001). Contemporary research has firmly established it as one of the key hallmarks of cancer development (Balkwill and Mantovani, 2001). Mechanistically, inflammation exerts multiple pro-tumorigenic effects. It stimulates angiogenesis and cell proliferation, inflicts DNA damage via reactive oxygen species, and suppresses cancer cell apoptosis (Takahashi et al., 2019).The tumor microenvironment, regulated by the inflammatory immune system, plays an important role in tumor proliferation and metastasis. The interaction between the tumor microenvironment-induced inflammatory response and the body’s immune status involves multiple mechanisms that can influence peripheral blood circulating cells. Therefore, peripheral blood cell counts and ratios can serve as indicators of the tumor microenvironment’s status. When there is an imbalance between the anti-tumor immune system and the inflammatory immune system, tumor-produced inflammatory mediators, such as tumor-necrogen-α are increased, leading to neutrophilia, thrombocytosis, and lymphocytopenia (Turan et al., 2018). The changes in blood routine parameters, including hemoglobin and platelet levels, have also been closely related to tumor development (Peila and Rohan, 2020; Ning et al., 2021). As shown in Figure 4, NPR with the cutoff of 0.024 had good predicted value for severe PTR (P = 0.01). In Table 4, we analyzed the association of clinical characteristics with severe PTR. By univariate and multivariate analysis, patients with age <60 years old had significantly higher severe PTR (90% VS 52.4%, P < 0.05), and patients with NPR <0.024 had significantly higher severe PTR (75.9% VS 41.2%, P < 0.05). Furthermore, we combine NPR and CPS to predict severe PTR in Table 5. In group of CPS 20–100, patients with NPR <0.024 could achieve a higher severe PTR (100% VS 54.5%, P = 0.004). In group of CPS 2–19, patients with NPR <0.024 still had a significantly higher severe PTR (71.4% VS 20.0%). Even CPS <1, patients with NPR <0.024 still had a higher severe PTR. In clinical practice, when CPS is ≥1, ICIs can be considered for neoadjuvant therapy in HNSCC due to the favorable pathological response. When the CPS <1, it can be combined with NPR. If the NPR <0.024, ICIs can be applied; otherwise, it should be considered carefully on a case-by-case basis.

Nevertheless, our study has some limitations that should be addressed. The overall cohort was small. There was a predominance of patients with oropharyngeal and hypopharyngeal cancers and patients with oral and laryngeal cancers were limited, which may introduce potential bias. They should be validated in large sample sizes or multi-center studies. Further, the heterogeneity in the different types of immunological agents used may introduce variability in the results.

## Conclusion

Higher CPS can be considered a good predictor of higher PCR after NICC in patients with LAHNSCC. Patients with CPS <1 can still achieve a higher PTR. Patients with NPR <0.024 can help achieve a higher severe PTR in patients with LAHNSCC regardless of the CPS.CPS combined with NPR may have a better predicted value for surgical PR of HNSCC after NICC.

## Data Availability

The raw data supporting the conclusions of this article will be made available by the authors, without undue reservation.
